# Integrating Health Technologies in Health Services for Syrian Refugees in Lebanon: Qualitative Study

**DOI:** 10.2196/14283

**Published:** 2020-07-06

**Authors:** Reem Talhouk, Chaza Akik, Vera Araujo-Soares, Balsam Ahmad, Sandra Mesmar, Patrick Olivier, Madeline Balaam, Kyle Montague, Andrew Garbett, Hala Ghattas

**Affiliations:** 1 School of Design Northumbria University Newcastle upon Tyne United Kingdom; 2 Open Lab Computing Newcastle upon Tyne United Kingdom; 3 Center for Research on Population and Health Faculty of Health Sciences American University of Beirut Beirut Lebanon; 4 Institute of Health and Society Faculty of Medical Sciences Newcastle University Newcastle upon Tyne United Kingdom; 5 Faculty of Information Technology Monash University Melbourne Australia; 6 KTH Royal Institute of Technology Stockholm Sweden; 7 Computer and Information Sciences Northumbria University Newcastle upon Tyne United Kingdom

**Keywords:** Syrian refugees, Lebanon, health technologies, eHealth, mHealth, primary health care

## Abstract

**Background:**

Lebanon currently hosts around one million Syrian refugees. There has been an increasing interest in integrating eHealth and mHealth technologies into the provision of primary health care to refugees and Lebanese citizens.

**Objective:**

We aimed to gain a deeper understanding of the potential for technology integration in primary health care provision in the context of the protracted Syrian refugee crisis in Lebanon.

**Methods:**

A total of 17 face-to-face semistructured interviews were conducted with key informants (n=8) and health care providers (n=9) involved in the provision of health care to the Syrian refugee population in Lebanon. Interviews were audio recorded and directly translated and transcribed from Arabic to English. Thematic analysis was conducted.

**Results:**

Study participants indicated that varying resources, primarily time and the availability of technologies at primary health care centers, were the main challenges for integrating technologies for the provision of health care services for refugees. This challenge is compounded by refugees being viewed by participants as a mobile population thus making primary health care centers less willing to invest in refugee health technologies. Lastly, participant views regarding the health and technology literacies of refugees varied and that was considered to be a challenge that needs to be addressed for the successful integration of refugee health technologies.

**Conclusions:**

Our findings indicate that in the context of integrating technology into the provision of health care for refugees in a low or middle income country such as Lebanon, some barriers for technology integration related to the availability of resources are similar to those found elsewhere. However, we identified participant views of refugees’ health and technology literacies to be a challenge specific to the context of this refugee crisis. These challenges need to be addressed when considering refugee health technologies. This could be done by increasing the visibility of refugee capabilities and configuring refugee health technologies so that they may create spaces in which refugees are empowered within the health care system and can work toward debunking the views discovered in this study.

## Introduction

Lebanon currently hosts around one million Syrian refugees [1]. This influx of refugees has resulted in a 30% increase in the country’s total population size, placing a burden on the Lebanese health care system [2]. However, despite political and social turmoil and a shortage in international financing and support in addressing the refugee crisis, the Lebanese health care system has proven to be resilient through coordinating efforts of various stakeholders (international and local) and maintaining a diverse health system while strengthening infrastructure and health and human resources [3]. As a result, refugee health care provision has been integrated into the Lebanese health care system with primary health care centers (PHCs) as the first point of entry into the health care system [3].

Technology that facilitates the transmission of health-related information (eHealth) [4] has been considered by the World Health Organization to be an effective tool that improves equity and quality of health care provision while reducing costs [5]. As such, it is argued that eHealth is an enabler of achieving universal health coverage, which is part of the agenda of meeting Sustainable Development Goal 3 to “ensure healthy lives and promote well-being for all at all ages” [5]. More specific to the context of refugees, eHealth has been found to aid in the monitoring and evaluation of refugee health statuses during times of protracted crises [6].

The increasing ubiquity of technologies among refugee communities has created a space in which refugee communities may engage with aid services through technologies [7]. Interviews with over 100 Syrian refugees in Lebanon found that most interviewees have access to a cell phone, of which 40% had smartphones [8]. A study with Syrian refugees in Lebanon [9] identified that refugees in informal tented settlements in rural Lebanon have access to at least one smartphone per household and access the internet through a Wi-Fi network set up in the settlement. Furthermore, Talhouk et al [9], through a series of focus groups, found that Syrian refugees in rural Lebanon are highly motivated to use smartphones as a means of communicating with health care providers and accessing health services provided by PHCs.

Several projects in Lebanon have begun integrating eHealth and mHealth, a category of eHealth technologies that use mobile phones for health services [10], to strengthen PHCs and improve the delivery of health care services to Syrian and Palestinian refugees as well as to Lebanese citizens. One initiative aimed to improve quality of care delivered by providing health care providers with electronic learning materials allowing for peer-to-peer exchanges through online forums [11,12]. An mHealth app for refugees was also piloted with clinicians in PHCs in Lebanon as a means of improving the provision of services to refugees suffering from chronic diseases and was shown to improve a number of quality of care indicators [13]. It is important to note that the mHealth tool used patient’s cell phone number as an identifier in order to ensure that clinicians can access patient medical records if patients change locations [13].

Saleh et al [14] conducted a randomized controlled trial (RCT) with PHCs in rural Lebanon and in Palestinian refugee camps in which short message service (SMS) messages communicating medical information, importance of compliance, and appointment reminders were sent to participants in the intervention group and were shown to significantly improve blood pressure control and hemoglobin A_1C_ levels used for the diagnosis and monitoring of diabetes. However, this RCT also indicated that SMS had no significant impact on patients’ use of primary health care services [14]. Also in collaboration with PHCs in rural Lebanon and Palestinian refugee camps, a netbook application was piloted as an eHealth tool to support community health workers in screening community members for diabetes and hypertension and subsequently referring them to primary health care clinics [4]. The study highlighted the ability of eHealth community-based interventions to identify new cases of diabetes and hypertension and refer them to the appropriate health services [4].

Low levels of agency in the Syrian refugee population in Lebanon and their low level of access and use of antenatal care services has also been the subject of mHealth interventions. Talhouk et al [15] piloted community health radio shows through mobile calls that delivered health information and responded to refugees’ reproductive health questions. These interactions around reproductive health led to an increase in refugee agency and trust within patient-provider relationships [15]. A quantitative study conducted with primary health care providers across 22 PHCs in Lebanon showed high health care provider readiness to adopt eHealth and encouraged the Lebanese Ministry of Public Health (MoPH) and policy makers to enhance and scale up eHealth initiatives within the primary health care system [18]. Additionally, based on their success in piloting eHealth and mHealth technologies in PHCs run by the MoPH and the United Nations Relief and Works Agency for Palestine Refugees in the Near East [4,14], the authors called for the scaling up of mHealth and eHealth interventions to the wider PHC network in Lebanon (ie, PHCs funded and managed by other health care organizations).

While research has been conducted on eHealth systems for refugees in Lebanon, no study provides a qualitative perspective that engages with multiple stakeholders on the potential of implementing eHealth for Syrian refugees in Lebanon with the aim of meeting SDG3. Indeed, the existing studies to date do not explore the added considerations needed to integrate eHealth technologies into primary health care services specifically for refugees. We present a qualitative study to gain a deeper understanding of the potential for technology integration in the provision of primary health care in the context of the protracted Syrian refugee crisis in Lebanon. We use reproductive and maternal health care as an entry point as this research is part of a wider project on technologies for refugee women’s health. The findings from interviews with participants highlighted factors that need to be considered when implementing eHealth projects, such as (1) varying resources available at PHCs, (2) mobility of refugee populations, and (3) varying views of refugee technology and health literacies. In our discussion, we provide practical and theoretical implications for integrating eHealth technologies for refugee health care that include countering views regarding refugee health and technology literacies.

## Methods

### Data Collection

We recruited key informants and health care providers involved in the provision of reproductive and maternal health services to refugees. Participants spanned the humanitarian health response system both vertically (from policy makers to clinical staff) and horizontally (participants from multiple organizations). Key informants were identified from meeting minutes published on the United Nations High Commissioner for Refugees Regional Refugee Response Interagency portal for Lebanon [1]. Potential participants were approached via email, and a convenience sample was drawn based on responses (response rate: 80%). Three PHCs catering to large numbers of Syrian refugees were purposively selected. The PHCs were situated in the West Bekaa region of Lebanon, and the centers varied in terms of management and affiliation with the MoPH primary health care network. Participants consisted of a convenience sample of health care providers available at the PHCs on the days of the research visit (response rate: 100%). Recruitment was completed once data saturation was met.

A total of 17 face-to-face semistructured interviews lasting around an hour each were conducted with key informants (n=8) and health care providers (n=9) involved in the provision of health care to the refugee population (Table 1).

Data were collected between January and March 2015. Ethical approval was obtained from the institutional review board of the American University of Beirut and Newcastle University’s ethics committee. All interviews were conducted in Arabic by local coauthors with a background in health system management and policy at the workplace of interviewees.

**Table 1:** Description of study participants detailing the gender and role of participants within the humanitarian health system. Participant code presented is used in the reporting of results.

Participants		Number	Gender	Participant code
**Key informant**				
	Public sector employees	2	Male (1), female (1)	Key informant 1,2
	Health center directors	2	Male (2)	Key informant 3,4
	Academics	2	Male (1), female (1)	Key informant 5,6
	Nongovernmental employees	2	Female (2)	Key informant 7,8
**Health care provider**				
	Nurses	5	Male (2), female (3)	Nurse 1-5
	Medical doctors	2	Male (2)	Doctor 1,2
	Social workers	2	Female (2)	Social worker 1,2

### Key Informant Interviews

Key informant interviews were conducted with PHC directors, nongovernmental organization employees, public sector employees, and academics working on eHealth within the Lebanese health care system. It is important to note that both PHC directors were also physicians. We probed on the current technologies being used, participant views on technology use, possible future use of technology, and willingness of their organizations to support the use of technology. This involved ascertaining equipment availability and organizational technological literacy. Interviewees were then asked to brainstorm about possible technologies that may be useful and the feasibility of these technologies within their current context.

### Health Care Provider Interviews

Nurses, doctors, and social workers constituted this subset of study participants. In these interviews, questions focused on the day-to-day processes and challenges of health service provision to Syrian refugees and the interactions between health care providers and Syrian refugees. Interactions explored included those within the PHC, those within the community, and those conducted through technology. We also explored the ways in which health care providers are currently using technology, both formally and informally, and their comfort and willingness to use different forms of technology for refugee health care provision.

### Data Analysis

Simultaneous translation, from Arabic to English, and transcription of the audio-recorded interviews was undertaken. Thematic analysis was conducted using NVivo 10 (QSR International) by two of the coauthors [16]. Codes were then grouped into themes and validated by a third coauthor. Data collection was concluded once data saturation was met and no new themes were emerging from the (data) interviews.

## Results

### Findings

Our analysis identified differences in levels of resources available across PHCs to be a key factor that needs to be considered when integrating and scaling a health technology within the primary health care system. Additionally, the mobility of refugee communities was found to be a disincentive consideration for the integration of technologies specifically for refugee reproductive health. Lastly participants recounted experiences of engaging with refugees in which they identified varying views regarding refugee technology and health literacies. Such views are key considerations that should be accounted for when designing technologies that specifically connect refugees to the health care system.

### Varying Resources Available at Primary Health Care Centers

A key consideration that needs to be accounted for when considering integrating and scaling up technologies within the refugee health care response system is the shortage in human resources as reported by key informant 2.

...they [center staff] might tell you we want to invest in more staff than in smartphones because we have a shortage in staff.

Key informant 2

In only one out of the three health centers visited by the research team did participants not identify shortages in staff and equipment as issues. Staff shortage was compounded by the increase in patient load due to the influx of Syrian refugees, resulting in nurses having to take work home with them.

[Since the refugee crisis began] in a short time my working hours increased [by] 6 hours, which then doubled and tripled.

Nurse 3

High patient load led to time constraints that discouraged the use of available health information systems (HISs).

We do not always have the time [to use the HIS]. We do a hundred things at the same time.

Nurse 2

Limited equipment available was also indicated to be a barrier to the use of existing technologies in PHCs.

We both are using one laptop...when she is working on the laptop, I cannot work on it... I have to enter 400 files and I’m 6 days behind... The time she [my colleague] spends on the laptop is time lost from my work.

Nurse 3

The main issue is [we need] to have more computers. All the work is being assigned to me [because there is one laptop] and I have to take the work home sometimes when this is not necessary.

Nurse 1

Interviews conducted at the PHC, which had an advanced HIS and was well resourced, revealed that shortage of resources was not a barrier for the integration of technology. The varying resources available at the different PHCs were attributed to the diversity of stakeholders involved in the health care provision of refugee reproductive health services. While some of the PHCs were part of the MoPH’s primary health care network, others were not.

The current process is that we are working with MoPH but not all primary health care centers are within the network. Not all PHCs are supported by the MoPH.

Key informant 2

This resulted in varying levels of support regarding the health center management and funding received as well as the use of varying eHealth systems. Interviewees reported that while several health centers were in the process of transitioning at the time of data collection to the use of a new HIS designed and supplied by the MoPH, other health centers were not as they have their own systems. In fact, the MoPH was reported to be incentivizing PHCs to adopt health information technologies by adding them to their accreditation standards and making HIS integration a key compliance criterion for MoPH contracting.

[Implementation of] the information system is a main criterion of the [MoPH] contract with PHCs. The centers can use their own system or the MoPH’s.

Key informant 2

However, both the contracts and accreditation are not extended to PHCs outside the MoPH primary health care network. The MoPH is also encouraging the use of HIS by providing training for health care providers on the system that they have developed. An interviewee indicated that continuous training is needed due to the high staff turnover at the PHCs.

Training sessions are continuously held centrally with follows-ups at PHCs

Key informant 2

Academics working on an eHealth project in Lebanon stated that they were collaborating with health centers managed by one stakeholder to overcome the variations in human and technological resources available among multiple stakeholders.

### Mobility of Refugee Populations as a Disincentive to Investing in Refugee Health Technologies

The use of technology specifically for refugee populations was discouraged by participants when they considered refugees’ unstable political and physical environments.

We are facing a difficulty in this [using mobile technology] actually. They might have a phone but are afraid of giving [their number]. For [security] reasons they don’t give you the real number.

Doctor 2

Now regarding maintaining it [the technology], it might be low because they may go from one place to another or go back to Syria or change the location of the tents. You know things are happening, like a fire in a tent or the flooding of a tent. Forces of nature are impacting them a lot so they are [moving] quickly.

Social worker 1

Pilot eHealth interventions have excluded Syrian refugees due to their mobility.

Because they are a mobile population. For ethical reasons if a community health worker encounters Syrian refugees, they will be screened [for chronic diseases] but not included in our database.

Key informant 6

A PHC director attributed his hesitance in investing in using a technological system to improve the provision of health care for refugees to the high mobility of the community and their unstable presence in Lebanon.

So let us say we make something advanced and then they leave; everything we have done would be lost.

Key informant 3

### Varying Views of Refugee Technology and Health Literacies

Participants recounted experiences of engaging with refugees that resulted in varying views regarding refugee technology and health literacies. Participants highlighted that the low level of education among refugees limits their ability to use technologies as well as actively engage with the health care system. This notion was disputed by two participants who indicated that refugee women they are encountering are highly educated and highly concerned with their health and that incentivization may support refugees in engaging with health technologies.

### Experiences of Technology Literacy of Refugees

All participants except two questioned the ability of Syrian refugees, who were reported to be of lower technology literacies and not capable of engaging through digital technologies with service providers.

You don’t want to forget that they are of low educational status with all my respect for them. They [Syrians] have educated people but the pregnant women and the refugees...are of a low educational status... They would not know how to [create a voice] record as there is a lot of ignorance.

Nurse 5

A participant, a social worker who visits refugee settlements, contradicted this view and stated that technological interventions would be feasible given the high education among a large proportion of female refugees.

There are very few ignorant women. Did you know that most of the women in camps have finished their college education?

Social worker 1

### Experiences of Health Literacy of Refugees

Although participants reported that many Syrian refugees attended health centers to address their health concerns, they indicated that there was a lack of motivation among the refugee community to engage with ways that facilitated access and provision of health care. Participants used Syrian refugees’ lack of maintenance of medical papers and documentation as an analogy to refugees’ inability to maintain technologies. When brainstorming the feasibility of using a technology that embeds audio recording hardware into a health education book/health journal, several participants stated:

They don’t maintain a paper, I don’t think they would maintain a file.

Nurse 4

They barely maintain their ID; they bring it torn. Even the vaccination record they bring it ripped apart.

Key informant 1

Another participant indicated that refugees are health literate however incentives are needed to support them in adopting and participating in any technology that connects them to health care providers.

You should tell them...if you take care of it [the technology], you will be compensated somehow in the end.

Key informant 7

## Discussion

### Principal Findings

Our findings indicate that in the context of integrating technology into the provision of health care for refugees in a low or middle income country such as Lebanon, some barriers for technology adoption are common with those found elsewhere [17]. However, certain factors that hinder the use of technology were found to be specific to the provision of refugee health care. Such distinctions have not been made in previous studies assessing the eHealth readiness of Lebanese primary health care centers [18] nor in the literature on the use of technologies to improve refugee health [4,13,14].

The multitude of stakeholders involved within the underresourced health care system poses a huge barrier for the consistent use of technology across the primary health care system. Additionally, participants indicated time constraints to be a major factor that contributes to the perception of technology as a burden within health centers. Such barriers are consistent with literature on technology integration into health care systems [19] and with studies conducted in Lebanon where mHealth technologies situated in PHCs were found to be redundant given the presence of parallel technological systems already in use in the PHCs [13]. Additionally, previous eHealth and mHealth [4,13,14] studies in Lebanon have been primarily conducted with one organization managing multiple PHCs thus providing limited insight on the barriers of scaling up such technologies to the wider primary health care system. Within such contexts, Khalifehsoltani et al [19] recommends practical implications that constitute a top-down policy approach as a means of overcoming barriers to the integration of health technologies introduced by the fragmentation and underresourcefulness of the health care system. For successful eHealth integration in low and middle income countries, policies should be put in place that strengthen the government’s capacity to plan, manage, regulate, and enforce eHealth policies in a way that incentivizes or mandates third party actors to adopt eHealth technologies [19]. Furthermore, a top-down approach to eHealth integration would allow for the exploration of interoperability of programs within health care systems that include a multiplicity of stakeholders [17,19]. Yassin et al [10] have indicated the Lebanese MoPH has begun introducing policies to encourage the uptake of eHealth and mHealth technologies in PHCs in a manner that would aid in overcoming the systematic challenges identified in our study.

Moreover, our findings provide theoretical contributions through highlighting that when it comes to integrating technologies for the provision of health care for refugees specifically, new factors are at play which require further consideration. Participants justified the unfeasibility of using technology within refugee health care based on their view of refugees as a mobile community that is of low health and technology literacy. Such notions were disputed by other participants who viewed refugees to be of high literacy and in need of support to engage in health technologies. Such factors have not been previously reported within literature on integrating eHealth.

In light of such evidence, practical implications include raising awareness among health care providers and PHC management that existing research indicates that Syrian refugees in Lebanon have high technological and health literacies [8,9,20] and creating spaces in which refugees may practice and demonstrate their technological and health literacies. Interactions between health care providers and refugees mediated through technological interventions themselves may create spaces in which refugees are empowered within the health care system and can work toward debunking the negative views surfaced in our study. Virtual nurse agents were explored by Bickmore et al [21] as a means of empowering low health literacy hospital patients, while medical information visualization has been used to better inform patients of their medical conditions and consequently facilitate their communication with health care providers and inform decision making processes [22]. Such technologies aim at empowering patients by providing them with information regarding their health issues. Other technologies aim to empower patients in the health care system by facilitating their offline communications with health care providers. Jacobs et al [23] and Mirkovic [24] highlighted the potential for health technologies to allow cancer patients to prioritize the issues they would like to discuss during their face-to-face engagements with health care providers, while Colley et al [25] introduced a dual-sided tablet to be used during clinical consultation where one side of the tablet provides constructive information to the patient regarding what the doctor is inputting.

Technological designs may allow us to reconfigure the current modalities of communication to allow refugees to take on a more proactive role within their health care rather than being just recipients of a service [26]. Indeed, technology may be situated within refugee communities where they could initiate the use of the technology themselves, in order to communicate with health care providers and/or other parts of the humanitarian system. Such an approach would overcome the barriers of refugee mobility by giving refugees the possibility of initiating contact with the health care system regardless of where they are. Moreover, such a reconfiguration may play a role in changing the views of health care providers toward refugees by creating more open communication mediums where refugees can demonstrate their technological literacy and health agency [15]. Previous work with Syrian refugees has shown that such configurations mediated through refugee-led community radio shows provide health care providers with a deeper understanding of the refugee community and enable refugees to vocalize their health concerns [15].

### Strengths and Limitations

This study provides an understanding of the barriers to integrating refugee health technologies in underresourced health systems such as Lebanon. The study was conducted in the West Bekaa region where a large number of refugees live; however, it may not represent the realities of other geographies in this context. Further research is thus needed in order to ascertain the generalizability of the results. Additionally, future studies and eHealth endeavors in similar contexts should actively engage with technology designers and developers who are stakeholders playing key roles in the configuration and deployment of health technologies.

The diversity of participants in this study reflects the different stakeholders and actors who engage in the provision of primary health care for refugees in Lebanon. The selection of a range of different stakeholders and actors ensures that the varying perspectives are reflected and information can be triangulated for reliability and validity. Our findings highlight factors that need to be further investigated before the implementation of eHealth for refugees.

### Conclusions

The introduction of technologies into health care systems in underresourced countries for the provision of health services to refugee communities is likely to face barriers similar to those we identified in this study, particularly in contexts with fragmented and underresourced health systems. Our study provides theoretical contributions by highlighting barriers specific to how refugees are perceived by health care providers and actors within the health care system. While there is evidence that may disqualify such views, the technological literacy and health motivation of refugees should be made more visible to health care providers and other actors within the system. Technologies that empower refugees when accessing health care and those that challenge the current modalities in which refugees communicate with health care providers could play a role in debunking such perceptions in the future.

## Abbreviations

eHealth: electronic health
EPSRC: Engineering and Physical Science Research Council
HIS: health information system
mHealth: mobile health
MoPH: Ministry of Public Health
PHC: primary health care center
RCT: randomized controlled trial

## References

[1] (2019). Figures at a glance. United Nations High Commissioner for Refugees.

[2] Ammar W, Kdouh O, Hammoud R, Hamadeh R, Harb H, Ammar Z, Atun R, Christiani D, Zalloua PA (2016). Health system resilience: Lebanon and the Syrian refugee crisis. J Glob Health.

[3] Ammar W, Radi A, El-Jardali F (2016). Comments on the article: Syrian refugees in Lebanon: the search for universal health coverage. Confl Health.

[4] Saleh S, Alameddine M, Farah A, El Arnaout N, Dimassi H, Muntaner C, El Morr C (2018). eHealth as a facilitator of equitable access to primary healthcare: the case of caring for non-communicable diseases in rural and refugee settings in Lebanon. Int J Public Health.

[5] Al-Shorbaji N (2018). Telemedicine. QJM An Int J Med.

[6] Mesmar S, Talhouk R, Akik C, Olivier P, Elhajj I, Elbassuoni S (2016). The impact of digital technology on health of populations affected by humanitarian crises: recent innovations and current gaps. J Public Health Policy.

[7] Maitland C (2018). Introduction. Digital Lifeline? ICTs for Refugees and Displaced Persons.

[8] (2013). Lost: Syrian refugees and the information gap. Internews.

[9] Talhouk R, Mesmar S, Thieme A, Balaam M, Olivier P, Akik C (2016). Syrian refugees and digital health in lebanon: opportunities for improving antenatal health. Proc CHI Conf Human Factors Comput Syst.

[10] Yassin N, Khodor R, Baroud M, Braithwaite J, Mannion R (2018). m-Health for healthcare delivery reform: prospects for Lebanese and refugee communities. Healthcare Systems: Future Predictions for Global Care.

[11] Hanafy S (2017). Digital health platforms to improve health of Syrian refugees in Lebanon. Int J Health Sci.

[12] Saleh S, Sinha C (2016). Using digital tech to improve life for refugees. International Development Research Centre.

[13] Doocy S, Paik KE, Lyles E, Hei TH, Fahed Z, Winkler E, Kontunen K, Mkanna A, Burnham G (2017). Guidelines and mHealth to improve quality of hypertension and type 2 diabetes care for vulnerable populations in Lebanon: longitudinal cohort study. JMIR Mhealth Uhealth.

[14] Saleh S, Farah A, Dimassi H, El Arnaout N, Constantin J, Osman M, El Morr C, Alameddine M (2018). Using mobile health to enhance outcomes of noncommunicable diseases care in rural settings and refugee camps: randomized controlled trial. JMIR Mhealth Uhealth.

[15] Talhouk R, Bartindale T, Montague K, Mesmar S, Akik C, Ghassani A (2017). Implications of synchronous IVR radio on Syrian refugee health and community dynamics. Proc 8th Int Conf Communities Technol.

[16] Braun V, Clarke V (2006). Using thematic analysis in psychology. Qual Res Psychol.

[17] Gerber T, Ranck J (2016). eHealth policy in LMICS: national frontiers, global challenges. Disruptive Cooperation in Digital Health.

[18] Saleh S, Khodor R, Alameddine M, Baroud M (2016). Readiness of healthcare providers for eHealth: the case from primary healthcare centers in Lebanon. BMC Health Serv Res.

[19] Khalifehsoltani S, Gerami M (2010). E-health challenges, opportunities and experiences of developing countries. http://ieeexplore.ieee.org/document/5432436/.

[20] Talhouk R, Balaam M, Toombs A, Akik C, Ghattas H, Araújo-Soares V (2019). Involving Syrian refugees in design research: lessons learnt from the field. Proc 2019 Designing Interact Syst Conf.

[21] Bickmore T, Pfeifer L, Jack B (2009). Taking the time to care: empowering low health literacy hospital patients with virtual nurse agents. Proc SIGCHI Conf Human Factors Comput Syst.

[22] Rajwan Y, Kim G (2010). Medical information visualization conceptual model for patient-physician health communication. Proc Int Conf Health Informatics.

[23] Jacobs M, Clawson J, Mynatt E (2015). Comparing health information sharing preferences of cancer patients, doctors, and navigators. Proc Conf Computer Supported Cooperative Work Soc Comput.

[24] Mirkovic J (2013). Utilizing emerging technologies to promote more efficient face-to-face patient-clinician communication. Proc Conf Pervasive ubiquitous Comput.

[25] Colley A, Rantakari J, Häkkilä J (2015). Dual sided tablet supporting doctor-patient interaction. Proc Conf Companion Computer Supported Cooperative Work Soc Comput.

[26] Olivier P, Wright P (2015). Digital civics.

